# Effects of *Payena dasyphylla* (Miq.) on hyaluronidase enzyme activity and metalloproteinases protein expressions in interleukin-1β stimulated human chondrocytes cells

**DOI:** 10.1186/1472-6882-13-213

**Published:** 2013-08-23

**Authors:** Kamini Citalingam, Seema Zareen, Khozirah Shaari, Syahida Ahmad

**Affiliations:** 1 Department of Biochemistry, Faculty of Biotechnology and Biomolecular Sciences, Universiti Putra Malaysia, 43400 UPM Serdang, Selangor, Malaysia; 2 Faculty of Industrial Sciences and Technology, Universiti Malaysia Pahang, Lebuhraya Tun Razak, 26300 Gambang, Pahang, Malaysia; 3 Laboratory of Natural Products, Institute of Bioscience, Universiti Putra Malaysia, 43400 UPM Serdang, Selangor, Malaysia; 4 Department of Chemistry, Faculty of Sciences, Universiti Putra Malaysia, 43400 UPM Serdang, Selangor, Malaysia

**Keywords:** Osteoarthritis, Hyaluronidase, MMP-3, MMP-13, Payena dasyphylla

## Abstract

**Background:**

Hyaluronidases have been found as the target enzymes in the development of osteoarthritis (OA) disease. While there is still no curative treatment for this disease, recent studies on the treatment of OA were focused on the effectiveness of natural products which are expected to improve the symptoms with minimal side effects. The aim of this study was to screen selected Malaysian plants on their anti-hyaluronidase activity as well as to evaluate the active plant and its derived fractions on its potential anti-arthritic and antioxidant activities.

**Methods:**

A total of 20 methanolic crude extracts (bark and leaf) from ten different plants were screened using a colorimetric hyaluronidase enzymatic assay. The active plant extract (*Payena dasyphylla*) was then studied for its hyaluronidase inhibitory activity in the interleukin-1β (IL-1β) stimulated human chondrocytes cell line (NHAC-kn) using zymography method. The *Payena dasyphylla* methanolic bark extract was then fractionated into several fractions in where the ethyl acetate (EA) fraction was evaluated for its inhibitory effects on the *HYAL1* and *HYAL2* gene expressions using reverse transcription-polymerase chain reaction (RT-PCR) technique. While the MMP-3 and MMP-13 protein expressions were evaluated using western blot method. The phenolic and flavonoid contents of the three fractions as well as the antioxidant property of the EA fraction were also evaluated.

**Results:**

Bark extract of *Payena dasyphylla* (100 μg/ml) showed the highest inhibitory activity against bovine testicular hyaluronidase with 91.63%. The plant extract also inhibited hyaluronidase expression in the cultured human chondrocyte cells in response to IL-1β (100 ng/ml). Similarly, treatment with *Payena dasyphylla* ethyl acetate *(*EA) fraction (100 μg/ml) inhibited the *HYAL1* and *HYAL2* mRNA gene expressions as well as MMP-3 and MMP-13 protein expression in a dose dependent manner. *Payena dasyphylla* EA fraction has demonstrated the highest amount of phenolic and flavonoid content with 168.62 ± 10.93 mg GAE/g and 95.96 ± 2.96 mg RE/g respectively as compared to water and hexane fractions. In addition, the *Payena dasyphylla* EA fraction showed strong antioxidant activity with IC_50_ value of 11.64 ± 1.69 μg/mL.

**Conclusion:**

These findings have shown that *Payena dasyphylla* might contained potential phenolic compounds that inhibiting the key enzyme in osteoarthritis development, which is the hyaluronidase enzyme through interruption of *HYAL1* and *HYAL1* gene expressions. The degradation of cartilage could also be inhibited by the plant through suppression of MMP-3 and MMP-13 protein expressions. We also reported that the inhibitory effect of *Payena dasyphylla* on hyaluronidase activity and expression might be due to its anti-oxidant property.

## Background

Joint related diseases such as arthritis, contributes a large segment of an orthopaedic’s caseload. Arthritic diseases interfere with our daily activities as it causes enormous burdens in terms of pain, crippling and disability. Osteoarthritis (OA), the most common form of arthritis is the leading cause of chronic disability at older age. It is estimated that the prevalence of symptomatic knee OA in population above the age of 65 is 30 % and women suffer from knee OA two times higher than men. OA is characterized by a slow progressive degeneration of articular cartilage that leads to joint signs and symptoms, including changes at the subchondral bone and synovium. OA commonly affects the hands, spine, knees and hips [1] with other joints such as the wrist, elbows, and shoulders less frequently involve. While all tissues involving the synovial joint are affected, the degeneration of articular cartilage is the main pathological feature in this disease. The breakdown of articular cartilage in OA may start as a focal lesion and progressively extend to involve multiple components of the synovial joints such as alteration in the bone underneath the cartilage, development of marginal outgrowths, osteophytes and increased thickness of subchondral bone [2].

Research has proven that although age is the substantial risk factor for the account of this disease, other factors are also known to affect the progression of OA which includes obesity, mechanical factors such as trauma or injury to the joint [1] and genetic abnormalities [3]. However, the exact pathogenesis of this disease is still poorly understood. But latest detection methods showed that OA does not only result from the breakdown of articular cartilage due to aging and biomechanical factors, but is also caused by elevated levels of hydrolytic enzymes activity [4,5]. Hyaluronidase or hyaluronate glycanohydrolase (EC 3.2.1.35) is large neglected class of hydrolytic enzymes that belongs to the family of degradative endoglucosaminidase. This enzyme was found to be one of the most predominant glycosidase present during cytokine-induced ECM degradation associated with the synovial joint disease [6]. Hyaluronidase is also widespread in nature, which present abundantly in mammals, insects, leeches and bacteria [7,8]. Hyaluronan or hyaluronic acid (HA), the major building blocks of the matrix components in the ECM are degraded upon exposure to free radicals and hyaluronidases (predominantly HYAL1, HYAL2, HYAL3). Hyaluronidases break down HA excessively resulting in the degeneration and gradual loss of articular cartilage leading to OA.

Besides, destruction of the ECM in cartilage tissue is also caused by the locally produced matrix metalloproteinases (MMPs). MMPs are a group of proteases which consists of collagenases (e.g. MMP-1 and MMP-13), stromelysins (e.g. MMP-3), gelatinases and membrane type MMPs [9]. MMPs have been implicated in the biophysical degradation of ECM and the collagenous framework of articular cartilage. Activated MMPs are capable of cleaving most of the components in the ECM including type II collagen and aggrecan. Stromelysin-1 (MMP-3) cleaves proteoglycans (PGs), collagens, gelatins and link protein of aggrecan, whereas collagenase-3 (MMP-13) cleaves type II collagen and aggrecan at particular sites [10]. In cases of degenerative joint disease, such as OA, the expression and synthesis of both MMP-3 and MMP-13 are increased.

Currently, OA is being treated using conventional therapeutic interventions which include drug treatments and injectable agents such as glucocorticoids and hyaluronan. Drug therapies although costly, have extensively being used in mild to moderate cases where the use of drug treatments is to control pain and prolong disease progression. Although drug medication have proven to be effective in controlling pain, the prolong use of this drugs have been associated with serious adverse effects, mainly ulcer formation and gastrointestinal complications [11]. Hence, there appears to be a need for drugs with low toxicity and effective in the treatment of OA. Therefore, patients are turning increasingly to alternative medications.

The uses of natural products in treating such diseases have been gaining attention due to reasons such as they are found in nature and safer. Furthermore, report on incidences of adverse effects from consumption of natural products seems low as it may provide a much needed alternative for patients with long-term chronic OA [12]. Hence, in this project *Payena dasyphylla* a selected Malaysian tree locally known as “nyatoh” has been studied for its inhibitory effect on hyaluronidase enzyme activity and MMPs protein expressions as well as its anti-radical scavenging activity.

## Methods

### Plant materials and preparation of extracts

All plant materials were collected from the Sekayu forest reserve, Terengganu, east peninsular of Malaysia. The ten plant species were identified by botanist, Dr. Shamsul Khamis from Institute of Bioscience, Universiti Putra Malaysia (UPM) and the voucher specimen numbers of the collected plant samples were deposited in the Herbarium, Biodiversity Unit, Institute of Bioscience, UPM. The methanolic crude extracts of the ten plants (bark and leaf) were prepared using a standard extraction protocol. Briefly, samples was first cut into small pieces, dried under the shade, grounded and macerated in distilled methanol at room temperature for 48 hours. The extracts were filtered and the filtrates were collected in a conical flask and kept aside. The residue was again soaked in a fresh volume of methanol and the soaking process was repeated 6 times until clear filtrates were obtained. All the filtrates were then pooled and evaporated to dryness under reduced pressure. The extracts were labelled and the yields were recorded and stored at 4°C prior to use. Plant crude samples were dissolved in 100% DMSO at concentration of 100 mg/mL and stored at 4°C prior to experiments.

### Hyaluronidase assay

The preliminary screening for the 20 plant samples was conducted using the colorimetric hyaluronidase enzymatic assay. Hyaluronidase inhibitory activity was measured spectrophotometrically according to the Morgan-Elson method described by Reissig et al., [13] with some modifications. Briefly, the plant crude samples (100 μg/mL) dissolved in DMSO were mixed with 250 μL of 2.5 mg/mL hyaluronan (HA), which dissolved in phosphate buffer (pH6.4) at 37°C. Then, 100 μL of hyaluronidase (1600 U/mL) from bovine testis was added and the reaction mixture was incubated for 3 hours at 37°C. After the incubation period, 50 μL of boric acid was added to the reaction tube and boiled (100°C) for 15 minutes to stop the reaction. The boiling mixture was then placed on ice and 1 mL of p-dimethylaminobenzaldehyde (DMAB) solution was added. The reaction tube was then incubated for another 20 minutes at 37°C for the development of maximum colorization. The mixture was then transferred into a 96 well microtiter plate and the absorbance was read at 585 nm by using a microplate reader (SpectraMax, Plus 384, Molecular Devices, Inc., USA).

### Cell cultures

Normal human articular chondrocyte derived from the knee (NHAC-kn) were maintained in a special chondrocyte basal medium mixed with 5% fetal bovine serum, growth factors and supplements (0.2% R3-IGF-1, 0.5% bFGF, 0.1% transferrin, 0.2% insulin, 0.1% GA-1000) and grown in a humidified 5% CO_2_ incubator at 37°C. The cells were grown in a monolayer culture. Medium was changed every 2–3 days and the cells were passaged weekly. Cells of passage number 10–25 were used throughout the whole research.

### Zymography

Hyaluronidase expression in the conditioned-media of NHAC-kn cell culture was analyzed through HA-substrate zymography according to the protocol described by Guntenhoener et al., [14] with slight modification. The culture medium of 8 mL was concentrated 10-fold by a membrane filter (Sartorius Stedim Biotech, Germany) to obtain a detectable amount of hyaluronidase enzyme. Samples that were treated for 24 hours in the absence or presence of 100 ng/mL of human IL-1β were loaded and run on a 10% SDS-polyacrylamide gel containing a final concentration of 0.17 mg/mL hyaluronan. Following electrophoresis, the gels were incubated in 3% Triton-X 100 solution for 15 minutes with agitation. The gels were then transferred into 0.1 M sodium formate, pH 3.5 containing 0.15 M sodium chloride (hyaluronidase buffer). Rinsed twice with the buffer and then incubated at 37°C overnight on a rotating shaker. After the overnight incubation, the gels were rinsed with distilled water and stained in 0.5% Alcian blue solution for 1 hour and destained with 40% ethanol in 3% acetic acid. Solution was change once every hour until bands of hyaluronidase were observed. The bands were imaged using the Versadoc, Bio-Rad imaging system and the intensities of the bands were quantified using the Quantity One program (Bio-Rad).

### Reverse transcription-polymerase chain reaction (RT-PCR)

NHAC-kn cells were cultured, stimulated and treated in 25 cm^2^ cell culture flask for 6 hours. The cells were stimulated with 10 ng/ml of human IL-1β followed by *Payena dasyphylla* treatment and all the cells were harvested for total RNA extraction using RNeasy mini kit (Qiagen, USA). By using the One-Step RT-PCR kit (Qiagen, USA), the RNA samples were subjected to RT-PCR, with selective primers for *HYAL1* (5^’^AGCTGGGAAAATACAAGAACC-3’ and 5’- TGAGCTGGATGGAGAAACTGG-3’), *HYAL2* (5’- GAGTTCGCAGCACAGCAGTTC-3’ and 5’- CACCCCAGAGGATGACACCAG-3’) and GAPDH (5’- TGGTATCGTGGAAGGACTCAT-3’ and 5’- GTGGGTGTCGCTGTTGAAGTC-3’). Primers used were adapted from published gene sequences by Flannery et al., [15]. GAPDH functioned as the internal as well as loading control. The RT-PCR products were then electrophoresed on an agarose gel and viewed using the Versadoc, Bio-Rad imaging system.

### Western blotting

Total secreted proteins from 8 mL conditioned medium of the NHAC-kn cells were concentrated 10-fold by a membrane filter (Sartorius Stedim Biotech, Germany) to obtain detectable amount of proteins. Equal volume of protein (20 μL) samples were incubated for 3 minutes at 95°C in a loading buffer (5 μL), and were separated on a 10% SDS polyacrylamide gel. The gels were run at 40 mA (20 mA per gel, constant current) for 1 hour. After the separation was completed, the protein bands were electrophoretically transferred onto a polyvinylidene diflouride (PVDF) membrane using TE 77 SemiPhor Semi-dry Transfer Unit (Amersham Pharmacia Biotech, Amersham, UK). The transfer process was conducted at constant voltage setting (10 V) for exactly 60 minutes. Next, the membrane was incubated with a blocking solution PBST containing 5% serum for 1 hour at 37°C, and then probed with goat anti-human polyclonal antibodies against MMP-3 (1:1000) (Abcam, USA) and MMP-13 (1:1000) (Santa Cruz, USA) separately overnight. Thereafter, membrane was washed 3 times with three changes of 200 mL of PBST, with every wash lasting for 10 minutes. This was followed by subsequent incubation with donkey anti-goat IgG conjugated with horseradish peroxidase (HRP) (R&D Systems, USA) secondary antibody (1: 1000) for 1 hour at room temperature. After incubation, membranes were washed in five changes of 200 mL of PBST, 10 minutes for each wash. The bound antibody on the membrane was detected using an enhanced chemiluminescence detection system according to the manufacturer’s manual (Amersham Pharmacia Biotech, Amersham, UK).

### 2, 2-diphenyl-1-picrylhydrazyl (DPPH) radical scavenging assay

Measurements of radical scavenging assay were carried out according to the method described by Tagashira and Ohtake, [16] with slight modification. Stock solution of the sample was prepared at 100 mg/mL in methanol. The solution was diluted to different concentrations ranging from 100 to 1.56 μg/mL in a 96-well microtiter plate. Then, 5 μL of DPPH solution with the concentration of 1 mg/mL prepared in methanol was added to each well. The plate was then shaken gently and placed in dark for 30 minutes at room temperature. Ascorbic acid was used for comparison or as a positive control. The DPPH solution in the absence of the sample and contained only methanol was used as control. The absorbance was then measured at 517 nm using a microplate reader. Experiments were performed in triplicates. Percentage of DPPH free radical was calculated using the following equation:

$$\begin{array}{ll} \% & \mathrm{Radical}\;\mathrm{scavenging}\;\mathrm{activity}\\ & =\frac{{\mathrm{OD}}_{\mathrm{control}}{‒\mathrm{OD}}_{\mathrm{sample}}}{{\mathrm{OD}}_{\mathrm{control}}}\times 100 \end{array}$$

OD_control_ is absorbance from DPPH solution in methanol only and OD_sample_ is absorbance from DPPH solution treated with different concentration of sample.

### Phenolic content

The phenolic content of the sample was measured using Folin-Ciocalteu reagent based on procedure described by Singleton et al., [17] with some modifications. Stock solution of the sample was prepared at 100 mg/mL. Briefly, 50 μL of the solution was diluted with methanol to obtain the highest concentration 100 μg/mL in a 96-well microtiter plate. Then, the initial solution was mixed with 150 μL (1:10 v/v diluted with distilled water) Folin-Ciocalteau’s reagent and allowed to stand for 5 minutes at room temperature. Sodium carbonate (Na_2_CO_3_, 6%, w/v) at volume of 150 μL was added and the mixture was allowed to stand for another 90 min in the dark with intermittent shaking. The absorbance of the blue colour that developed was measured at 725 nm using a microplate reader. The experiment was carried out in triplicates. Gallic acid was used to construct the standard curve. A series of gallic acid concentration ranging from 25 μg/mL to 200 μg/mL was prepared in methanol and the phenolic compounds concentration in the sample was expressed as milligrams of gallic acid equivalent per gram of dry weight (mg GAE/g) of extract.

### Flavonoid content

The flavonoid content of the sample was determined based on procedure described by Zhishen et al., [18] with some modifications. Stock solution of the sample was prepared at 100 mg/mL. Briefly, 500 μL of the sample (100 μg/mL) was mixed with 2 mL of distilled water in a centrifuge tube. Then, 150 μL of aluminium trichloride (AlCl_3,_ 10% w/v) was added in and the reaction mixture was allowed to stand for 6 minutes at room temperature. Next, sodium nitrite (NaNO_2,_ 5% w/v) was added into the mixture and again was allowed to stand for 6 minutes, followed by addition of 2 mL of sodium hydroxide (NaOH, 4% w/v) into the same mixture. Subsequently, distilled water was added to bring the mixture up to final volume of 5 mL. Later, the reaction mixture was vortexed thoroughly and allowed to stand for another 15 minutes. The absorbance of pink colour that developed was measured at 510 nm using a microplate reader. The experiment was carried out in triplicates. Rutin was used to plot the standard curve. A series of rutin concentration ranging from 25 μg/mL to 200 μg/mL was prepared in methanol and the flavonoid content in the sample was expressed as milligrams of rutin equivalent per gram of extract (mg RE/g).

### Statistical analysis

The results were expressed as mean ± standard error of mean (SEM) from three independent experiments. Means and standard deviations were calculated from the data obtained and subjected to one-way analysis of variance (ANOVA) followed by Dunnett’s post-hoc test for multiple comparison with control. P values <0.05 were considered significant. The IC_50_ values were calculated using the statistical software package, GraphPad Prism 5.0 software.

## Results and discussion

### Screening of anti-hyaluronidase activity

Hyaluronidases have been targeted as one of the predominant glycosidase that is associated with osteoarthritis (OA) as it participates in the cartilage degradation mechanism [6]. Therefore, inhibition of hyaluronidase activity is crucial for normal metabolic processes to take place. Recent studies on the treatment and prevention of OA focused on the utility of botanical or natural products which is capable of ameliorating the symptoms of OA with expectation of limited side effects. Therefore, a total of ten species of selected Malaysian plants extracted in methanol on two parts (bark and leaf) were screened for their effect on hyaluronidase inhibitory activity using bovine testicular hyaluronidase. As presented in Table 1, the anti-hyaluronidase screening results showed that from the total of 20 methanolic crude extracts tested, three plants *Palaquium gutta, Pauteria obovatta* and *Payena dasyphylla* all extracted from the bark showed strong inhibitory activity against hyaluronidase with 88.82%, 90.47% and 91.63% respectively. This is followed by *Palaquium gutta* and *Pauteria obovatta* derived from the leaf plus *Uncaria villosa* from the bark which showed moderate inhibitory activity with 51.35%, 55.63% and 55.20% respectively. However, other plant extracts stated only weak inhibitory activity against hyaluronidase. Due to sample availability and highest inhibitory activity against hyaluronidase enzyme activity, only *Payena dasyphylla* was employed throughout the whole study.

**Table 1:** Effect of 10 selected Malaysian plants (bark and leaf) methanolic extracts on bovine testis hyaluronidase enzyme inhibitory activity

**No.**	**Plants**	**Family**	**Voucher/ Specimen No**	**Bark, % Inhibition**	**Leaf, % Inhibition**
1.	*Agalia densiflorum*	Meliaceae	SK 1562/08	26.14	14.17
2.	*Agalia macrophylla*	Meliaceae	SK 1563/08	38.71	2.41
3.	*Dyera costulate*	Apocynaceae	SK 1564/08	8.71	24.70
4.	*Gnetum cuspidatum*	Gnetaceae	SK 1566/08	35.00	8.26
5.	*Palaquium gutta*	Sapotaceae	SK 1572/08	88.82	51.35
6.	*Pauteria obovatta*	Sapotaceae	SK 1577/08	90.47	55.63
7.	*Payena dasyphylla*	Sapotaceae	SK 1565/08	91.63	24.10
8.	*Tabernae-montand dichotoma*	Apocynaceae	SK 1573/08	4.05	14.10
9.	*Uncaria acida*	Rubiaceae	SK 1570/08	37.32	10.50
10	*Uncaria villosa*	Rubiaceae	SK 1571/08	55.20	36.97

### Effect of *payena dasyphylla* methanolic extract on hyaluronidase enzyme activity in the cultured human chondrocyte cells

**Figure 1:**
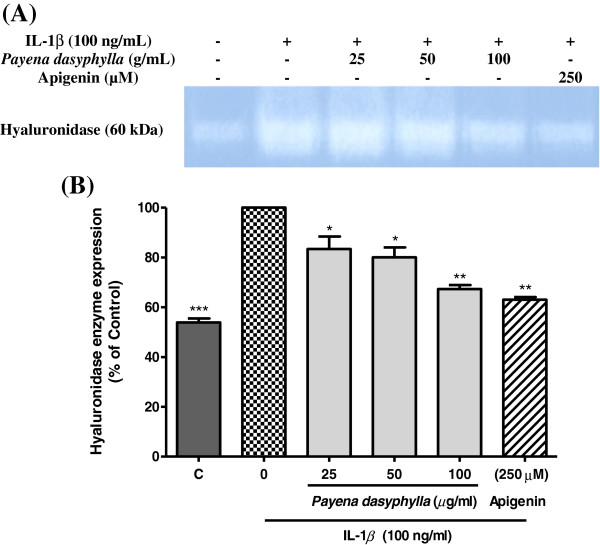
**Effect of *Payena dasyphylla *methanolic bark extract on the hyaluronidases enzymes expression.** Gel image **(A)** shows the zymography result represented from three independent experiments, while **(B)** shows the histogram of hyaluronidases enzyme from the same experiment. C; human chondrocytes cells (NHAC-kn) were cultured with medium alone (lane 1), cells induced with IL-1β (lane 2), cells induced and treated with *Payena dasyphylla* bark methanolic extract at 25, 50 and 100 μg/mL (lane 3,4 and 5 respectively) and cells induced with Apigenin (lane 6). Protein extracts prepared from the conditioned-medium of cultured NHAC-kn were subjected to SDS-PAGE co-polymerized with hyaluronan and electrophoresed. The gels were then stained with Alcian blue staining solution, destained and detected for cellular hyaluronidase expression. ***P < 0.001, **P < 0.01 and *P < 0.05 significantly different from IL-1β treated control group.

Chondrocyte cells were shown to exhibit the highest level of matrix anabolic and catabolic genes and thus, are preferred to be used in investigating chondrocytes destructive and synthesis activity as well as its regulation [19]. Besides that, human and bovine chondrocytes are the only cells present in articular cartilage that express hyaluronidases [15,20,21]. In this study, the effect of *P. dasyphylla* methanolic bark extract on cellular hyaluronidase expression was evaluated on the cultured human chondrocyte cell line (NHAC-kn). To mimic the degenerative effect of osteoarthritic cartilage, the cells were induced with human interleukin-1β (IL-1β). IL-1β is a pro-inflammatory cytokine that interferes with the extracellular matrix turnover by accelerating the degradation of cartilage matrix and inducing chondrocytes apoptosis. Results revealed that *P. dasyphylla* methanolic bark crude extract suppressed the hyaluronidase expression in the IL-1β stimulated chondroytes culture in a dose-dependent manner. This was observed through the decolouration (white band) of the blue background of the gel (Figure 1). Apigenin, a known hyaluronidase inhibitor *in vitro* was used as the positive control. Experimentally, this study demonstrates that treatment with human IL-1β has dramatically enhanced HA catabolism and treatment with *P. dasyphylla* has effectively inhibited the hyaluronidase expression from further degradation of HA.

Since *P. dasyphylla* methanolic crude extract showed effective in inhibiting hyaluronidase expression, it was thought worthwhile to fractionate the crude methanolic extract into several fractions based on the polarity of the compounds containing in the extract. *P. dasyphylla* crude extract was fractionated into three fractions using different range of solvents (water, ethyl acetate and hexane) whereby all the fractions were tested for its hyaluronidase inhibitory activity. It is determined that from the three fractions tested, Water fraction stated the highest inhibitory activity against hyaluronidase enzyme, followed by ethyl acetate (EA) fraction, and lastly the hexane fraction (results not shown). However, only EA fraction was chosen to be further evaluated on its effect toward the hyaluronidase enzyme. This is because, *P. dasyphylla* from the family of Sapotacea has been reported to contain high concentration of polyphenols such as tannins which usually contain in the water fraction and tannins are known to form complexes with a variety of proteins which may result in false positive results [22].

### Cytotoxicity effect of *Payena dasyphylla* bark extracts

**Figure 2:**
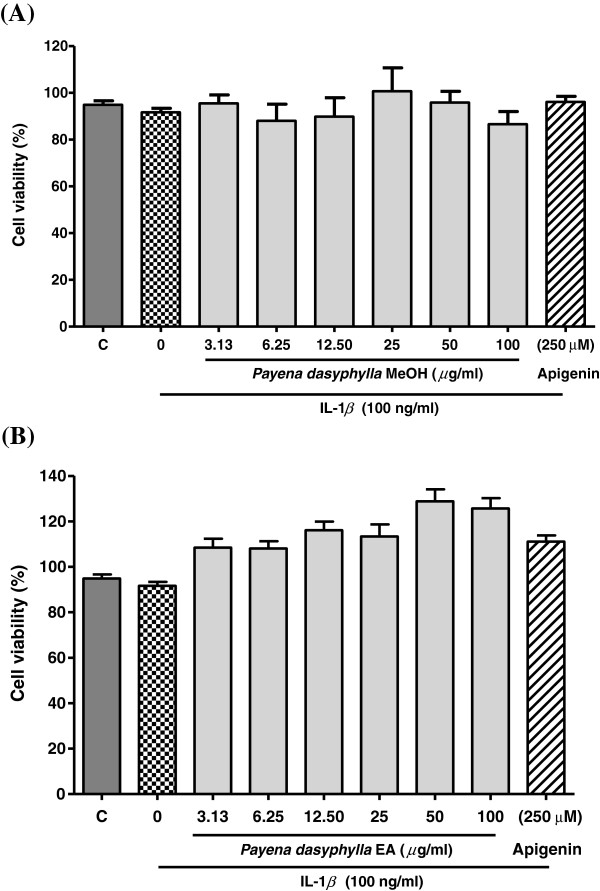
**Histogram shows above is the effect of (A) *Payena dasyphylla *methanolic bark extract and (B) *Payena dasyphylla *ethyl acetate (EA) bark fraction on NHAC-kn cell viability.** C; cells were cultured with medium alone and the rest are cells stimulated with 100 ng/ml IL-1β and treated with increasing concentration of plant extracts for 24 hours as indicated. The absorbance of MTT reduction was measured at a wavelength of 570 nm and the percentage of cell viability was measured against the control. The values are expressed as mean ± SEM in triplicates from three independent experiments (n = 3).

Nevertheless, the methanolic and EA fractions of *P.dasyphylla* were tested for their toxicity effect on the NHAC-kn cells induced with IL-1β. Results showed that both the extracts did not show any concomitant effect on the cells at all concentrations (Figure 2). In certain concentrations, the percentage of cell viability showed higher than 100% which could be due to the presence of bioactive principles in the extract that might have induced the proliferative effect of the cells [23]. Besides, it could also be due to nonuniform density of cell number at certain wells which could have affected the cell viability.

### Effect of *Payena dasyphylla* ethyl acetate (EA) fraction on the *HYAL1* and *HYAL2* mRNA gene expression

**Figure 3:**
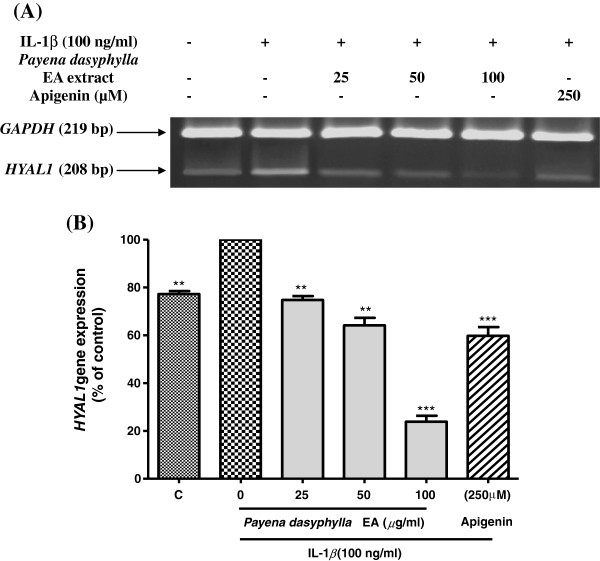
**Effect of *Payena dasyphylla *EA bark fraction on the *HYAL1 *gene expression in IL-1β stimulated NHAC-kn cells.** The gel image **(A)** shows the reverse transcriptase-PCR result represented from three independent experiments, while **(B)** shows the histogram of *HYAL1* gene expression from the same experiment. C; Basal level of *HYAL1* gene expression without IL-1β treatment and the rest are cells stimulated with 100 ng/ml human IL-1β with increasing concentrations of sample treatment for 6 hours. Apigenin (250 μM) was used as the positive control. Glyceraldehyde 3-phosphate dehydrogenase (GAPDH) gene expression was used as the loading control. ***P < 0.001 and **P < 0.01 significantly different from IL-1β treated control group.

**Figure 4:**
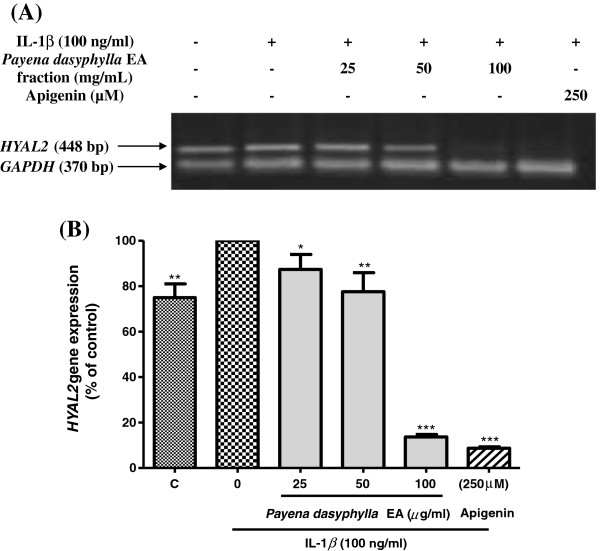
**Effect of *Payena dasyphylla *EA bark fraction on the *HYAL2 *gene expression in IL-1β stimulated NHAC-kn cells.** The gel image **(A)** shows the reverse transcriptase-PCR result represented from three independent experiments, while **(B)** shows the histogram of *HYAL2* gene expression from the same experiment. C; Basal level of *HYAL2* gene expression without IL-1β treatment and the rest are cells stimulated with 100 ng/ml human IL-1β with increasing concentrations of sample treatment for 6 hours. Apigenin (250 μM) was used as the positive control. Glyceraldehyde 3-phosphate dehydrogenase (GAPDH) gene expression was used as the loading control. ***P < 0.001, **P < 0.01 and *P < 0.05 significantly different from IL-1β treated control group.

It has been proposed that hyaluronan or hyaluronic acid (HA) catabolism occurs in a stepwise manner in where HA is first cleaved by Hyal2 into 20 kDa fragments which are then delivered into lysosomes for further degradation by Hyal1 and lysosomal *β*-exoglycosidases [24]. In order to determine whether *Payena dasyphylla* EA extract was able to inhibit the mRNA expression of the two principle hyaluronidases (*HYAL1* and *HYAL2*), RT-PCR analysis was utilized. Upon treatment with *P. dasyphylla* EA extract, results showed that *P. dasyphylla* inhibited the both genes, *HYAL1* and *HYAL2* in a dose dependent manner. Stimulation with IL-1β has up-regulated the gene expression of *HYAL1* (Figure 3) whereas for the case of *HYAL2* gene expression, there were notably little changes in the level of expression following treatment with IL-1β (Figure 4). This is because, *HYAL2* being predominantly expressed gene in the chondrocytes is showed to be constitutively expressed [21]. Therefore, stimulation with the inducer resulted in no remarkable changes in the expression. However, results from both the mRNA expression of the hyaluronidases shows that *HYAL1* and *HYAL2* are up-regulated by the treatment of IL-1β. This is in accordance with the study done by Nakamura et al., [25]. Findings from this data demonstrates that treatment with *P. dasyphylla* EA extract in response to human IL-1β has proven to be potential inhibiting the two principle hyaluronidases.

### Effect of *Payena dasyphylla* EA fraction on the MMP-3 and MMP-13 protein expression

**Figure 5:**
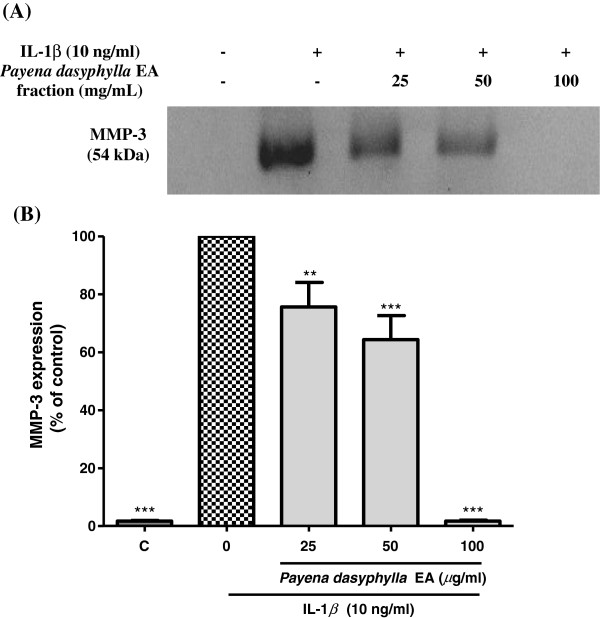
**Effect of *Payena dasyphylla *EA fraction on MMP-3 protein expression in IL-1β-stimulated NHAC-kn cells.** The blot image **(A)** shown above represents results from three independent experiments while **(B)** shows the histogram of MMP-3 protein expression from the same experiment. C; Basal level of MMP-3 expression without IL-1β treatment and the rest are cells stimulated with 10 ng/mL human IL-1β and treated with increasing concentrations of sample treatment for 24 h. Conditioned-medium were assayed for pretein expression by using Western blotting. ***P < 0.001 and **P < 0.01 significantly different from IL-1β treated control group.

**Figure 6:**
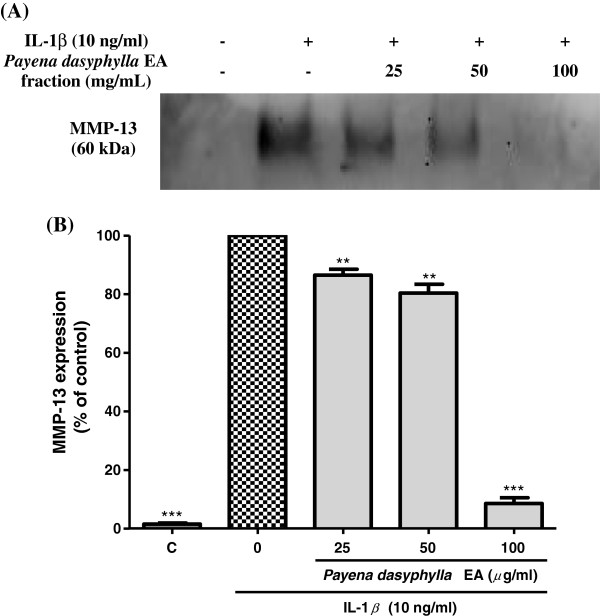
**Effect of *Payena dasyphylla *EA fraction on MMP-13 protein expression in IL-1β-stimulated NHAC-kn cells.** The blot image **(A)** shown above represents results from three independent experiments while **(B)** shows the histogram of MMP-13 protein expression from the same experiment. C; Basal level of MMP-13 expression without IL-1β treatment and the rest are cells stimulated with 10 ng/mL human IL-1β and treated with increasing concentrations of sample treatment for 24 h. Conditioned-medium were assayed for pretein expression by using Western blotting. ***P < 0.001 and **P < 0.01 significantly different from IL-1β treated control group.

Although hyaluronidases have been found to be the target enzyme in the development of osteoarthritic disease, studies have reported that MMPs also play a significant role in the destruction of the cartilage matrix in osteoarthritis [26]. MMP-3 and MMP-13 has assumed greater importance in OA because of its presence in human OA cartilage [27,28]. MMP-13 (collagenase) preferential degrades type II collagen whereas MMP-3 (stromelysin) degrades matrix components leading to the cleavage of collagen and proteoglycans [10,29,30]. Thus, the inhibitory effect of *P. dayphylla* EA extract on these two mediators was examined. Conditioned culture media derived from NHAC-kn cells treated with human IL-1β inducer showed that activated cells produced MMP-3 and MMP-13 protein expression [31,32]. Results showed that, in contrast to uninduced cells, no MMP bands were observed. However, treatment with *P. dasyphylla* EA extract in response to stimulation with human IL-1β inhibited the MMP-3 and MMP-13 expression suggesting that this plant extract is not selectively effective towards hyaluronidases, but is also potential against MMP-3 and MMP-13 expression (Figure 5 and Figure 6). Through IL-1β stimulation, MMPs production is significantly increased as this cytokine is involved in triggering the catabolism of cartilage degeneration. This finding is in agreement with other research done on rabbit and human femoral head chondrocytes where IL-1β was shown to induce expression of MMP proteins [29,31-35].

### Antioxidant capacity of *Payena dasyphylla* EA extract

**Figure 7:**
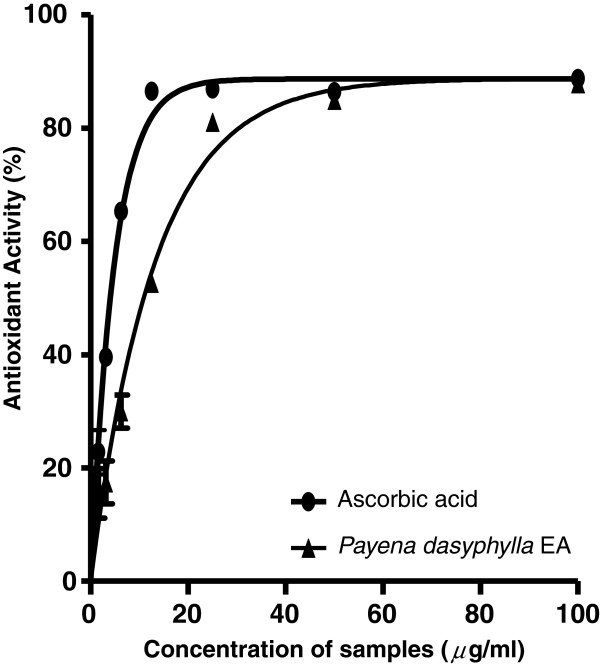
**Effect of *Payena dasyphylla *EA fraction and ascorbic acid on the antioxidant activity. *P. dasyphylla *showed 82.19% radical scavenging activity at highest concentration tested (100 μg/mL) with IC**_**50 **_**value of 11.64 ± 1.69 μg/mL.** Whereas, ascorbic acid exhibited antioxidant activity at 86.68% (100 μg/mL) with IC_50_ value of 4.03 ± 0.24 μg/mL.

Phenolic and flavonoid compounds are known secondary metabolites that are synthesized in plants. They are commonly found in both edible and inedible plants and have been reported to possess important biological effects such as antioxidant, anti-apoptosis, anti-aging, anticarcinogen, anti-inflammation, anti-artherosclerosis as well as inhibition of angiogenesis and cell proliferation [35-37]. Results showed that *P. dasyphylla* EA fraction showed the highest amount of phenolic and flavonoid content as compared to water and hexane fractions (Table 2). Whereas hexane fraction did not show any traces of flavonoid content. These results were consistent with other findings which had reported that hexanes (non-polar solvent) as well as water (polar solvent) were less efficient to extract phenolic compounds from plants [38-40]. Since flavonoid compounds have been reported to be potent metal chelators and free radical scavengers [41-44], the antioxidant capacity of this extract was also evaluated. Results showed that, at the concentration of 100 μg/mL, *P. dasyphylla* possessed 82.19% antioxidant radical scavenging activity with IC_50_ value of 11.64 ± 1.69 μg/mL. Ascorbic acid, a potent free radical scavenger was used as a positive control at a concentration of 100 μg/mL, exhibited 86.68% antioxidant activity with IC_50_ value of 4.03 ± 0.24 μg/mL (Figure 7). *P. dasyphylla* EA extract contained strong antioxidant property but not as high as compared to ascorbic acid, a known antioxidant. Studies have shown that free radicals play a part in promoting cartilage degradation in osteoarthritis [45]. Therefore, these finding suggested that phenolic compounds or flavonoids present in the fraction may have acted as the metal chelators responsible for its antioxidant activity.

**Table 2:** **Phenolic and flavonoid contents of *Payena dasyphylla *fractions**

***Payena dasyphylla***	^**a**^**Phenolic content**	^**b**^**Flavonoid content**
**fractions**	**(mg/g) GAE**	**(mg/g) RE**
Ethyl acetate	168.62 ± 10.93	95.96 ± 2.96
Water	84.32 ± 3.89	20.63 ± 0.87
Hexane	0.45 ± 0.02	-

Values are expressed as means ± SD of three independent experiments. ^a^GAE (gallic acid equivalents) and ^b^RE (Rutin equivalents).

## Conclusion

*Payena dasyphylla* is a local plant that has been extensively used in the timber industry. Till date, there have not been any studies reporting on its usage in the biomedical field. Our present study had demonstrated that *P. dasyphylla* possessed anti-arthritic property by inhibiting hyaluronidase enzyme activity and/expression by down regulating *HYAL1* and *HYAL2* gene expressions as well as MMP-3 and MMP-13 protein expressions in human chondrocytes cell line. In addition to its inhibitory effect, the ethyl acetate (EA) fraction of *P. dasyphylla* showed high content of phenolic or flavonoids which could be related to its radical scavenging power. Inhibition of these specific targets helps to restore and maintain the articular cartilage from further destruction in osteoarthritis. This knowledge may be useful for developing novel therapies for blocking IL-1β stimulated cartilage breakdown in osteoarthritis.

## Abbreviations

OS: (Osteoarthritis); IL-1β: (Interleukin-1beta); NHAC-kn: (Normal human articular chondrocytes from knee); MMP: (Matrix metalloproteinases); HA: (Hyaluronan); CO2: (Carbon dioxide); DPPH: (2, 2-diphenyl-1-picrylhydrazyl); ECM: (Extracellular matrix); EA: (Ethyl acetate); SDS-PAGE: (Sodium dodecyl sulphate-polyacrylamide gel electrophoresis).

## Competing interests

The authors declare that they have no competing interests.

## Authors’ contributions

KC conducted all the experiments, analyzed and interpretation of data and drafted the manuscript. SZ and KS were involved in the extraction and fractionation of the samples. SA was responsible for conception and design of the project, drafted the manuscript and revised it critically for important intellectual content. All authors read and approved the final manuscript.

## Authors’ information

KC was a Master’s student from the Faculty of Biotechnology and Biomolecular Sciences, Universiti Putra Malaysia (UPM), 43400 UPM Serdang, Selangor, Malaysia. SZ is a fellow researcher from the Faculty of Industrial Sciences and Technology, Universiti Malaysia Pahang, Lebuhraya Tun Razak, 26300 Gambang, Pahang, Malaysia. KS is an associate researcher at Laboratory of Natural Products, Institute of Bioscience and a professor at the Department of Chemistry, Faculty of Science, Universiti Putra Malaysia (UPM), 43400 UPM Serdang, Selangor, Malaysia. SA is a senior lecturer from the Department of Biochemistry, Faculty of Biotechnology and Biomolecular Sciences, Universiti Putra Malaysia (UPM), 43400 UPM Serdang, Selangor, Malaysia.

## Pre-publication history

The pre-publication history for this paper can be accessed here:

http://www.biomedcentral.com/1472-6882/13/213/prepub
